# Evaluating Measurement Bias in Flexural Modulus of Orthotropic UHMWPE Fiber/Nano-Epoxy Composites: A Finite Element Study

**DOI:** 10.3390/polym18212647

**Published:** 2026-10-26

**Authors:** Mehmet Vakkas Oğul, Halil İbrahim Yavuz, Umut Kaan Özbaş

**Affiliations:** Department of Materials Science and Nanotechnology Engineering, Faculty of Engineering and Natural Sciences, Yeditepe University, 26 Ağustos Campus, İnönü Mah., Kayışdağı Cad. 326A, Ataşehir, 34755 İstanbul, Türkiye; mehmetvakkas.ogul@std.yeditepe.edu.tr (M.V.O.); umutkaan.ozbas@std.yeditepe.edu.tr (U.K.Ö.)

**Keywords:** reactive graphitic nanofiber, nano-epoxy, UHMWPE fiber composite, three-point bending, span-to-depth ratio, transverse shear, finite element analysis, apparent flexural modulus

## Abstract

Reactive graphitic nanofibers (r-GNFs) dispersed in epoxy increase the resin’s flexural modulus by approximately 20% at a 0.3 wt% optimum loading, serving as matrices for ultra-high-molecular-weight polyethylene (UHMWPE) fiber composites. This study reanalyzes published datasets, constructing a traceable orthotropic material library evaluated in three-point bending via finite element analysis. The isotropic matrix cards successfully reproduce source experimental values, showing a maximum error of 0.50% at a 16:1 span and 0.020% at 32:1. However, for the unidirectional composite, the standard 16:1 span is predicted to yield an apparent flexural modulus of only 77.6–80.7% of the true longitudinal modulus. Crucially, this loss is formulation-dependent: r-GNFs increase the longitudinal modulus considerably more than the axial shear modulus, increasing the anisotropy ratio. Consequently, a 16:1 span underreports the true modulus gain at the optimum loading by 13%—an error that drops significantly when using a 32:1 span. Furthermore, we document issues in the source dataset: a lack of common fiber volume fraction across groups invalidates direct raw modulus comparisons, and the empirical model’s optimum coordinates appear transposed. Finally, a rule-of-mixtures decomposition confirms the 0.3 wt% optimum, revealing a statistically significant residual excess stiffness exclusively at this loading.

## 1. Introduction

Epoxy resins are widely used as matrices in structural composites because of their favourable mechanical and thermal properties and the absence of volatile emission during cure [1]. The addition of nanoscale fillers offers a route to further property improvement, and graphitic nanofibers (GNFs) are attractive in this context because they can be produced in large volume at low cost and because the exposed graphene edges allow chemical functionalization [2,3]. Functionalization of herringbone GNFs with 3,4′-oxydianiline yields reactive graphitic nanofibers (r-GNFs) in which reactive hydrogen participates in the epoxy curing reaction, producing a unified resin system rather than a simple physical mixture [3,4]. Jana et al. characterized the flexural behavior of r-GNF-reinforced epoxy using three-point bending in accordance with ASTM D790 [5] and reported that a loading of 0.3 wt% gave the highest flexural strength, modulus, and failure energy among all compositions tested, with a 26% increase in flexural strength and a 20% increase in flexural modulus relative to the neat resin [6]. The same research group subsequently used these nano-epoxy formulations as matrices for UHMWPE fiber bundle composites and reported tensile moduli as a function of both matrix volume fraction and r-GNF content, developing an empirical quadratic model relating the two [7]. The motivation for that work lies in the well-documented poor interfacial adhesion between the inert UHMWPE fiber surface and polymer matrices, which limits the overall mechanical performance of such composites [7,8].

Three-point bending is the standard method for assessing the flexural performance of polymeric materials, and both ASTM D790 and ISO 178 specify a default span-to-depth ratio of 16:1 [5,9]. The elementary beam relations:

(1) $$\sigma_{f}=\frac{3\mathrm{PL}}{2bd^{2}}$$

(2) $$\varepsilon_{f}=\frac{6\mathrm{Dd}}{L^{2}}$$

(3) $$E_{\mathrm{flex}}=\frac{PL^{3}}{48\mathrm{ID}}$$

where *σ_f_* is the flexural stress (MPa), ε_f_ is the flexural strain (-), *E*_flex_ is the flexural modulus (MPa), *P* is the applied load (N), *L* is the support span (mm), *b* is the specimen width (mm), *d* is the specimen depth (mm), *D* is the midspan deflection (mm), and *I* is the second moment of area, *I* = *bd*^3^/12 (mm^4^).

These relations are derived from the Euler–Bernoulli theory and therefore neglect transverse shear deformation. For isotropic polymers, where the ratio of Young’s modulus to shear modulus is approximately 2.6–2.8, this omission is inconsequential at 16:1. For unidirectional fiber composites, the situation is different: the longitudinal modulus is fiber-dominated, while the axial shear modulus is matrix-dominated, and their ratio can exceed 50. Under these conditions, the shear contribution to midspan deflection is no longer negligible, and the standards themselves acknowledge this by permitting longer spans for high-modulus materials [5].

What has not, to the authors’ knowledge, been quantified for this material system is the magnitude of the resulting error, and—more consequentially—whether that error is constant across formulations. If the filler modifies the longitudinal and shear moduli by different proportions, the anisotropy ratio itself becomes a function of filler content, and the measurement bias becomes formulation-dependent. In that case, the standard test does not merely shift all measured moduli by a fixed factor; it distorts the very quantity of interest, namely the relative improvement attributable to the filler.

The objectives of this work are therefore four-fold: (i) to construct a traceable, source-referenced material card library for both the nano-epoxy matrix and the UHMWPE fiber/nano-epoxy composite from the published datasets; (ii) to validate the matrix cards against the original experimental flexural moduli by finite element simulation; (iii) to quantify the apparent modulus loss in the composite at both the standard 16:1 and the extended 32:1 span ratio, using the isotropic matrix as a control; and (iv) to determine whether the r-GNF modulus gain reported by a standard flexural test differs from the true gain, and by how much.

## 2. Materials and Methods

**Figure 1:**
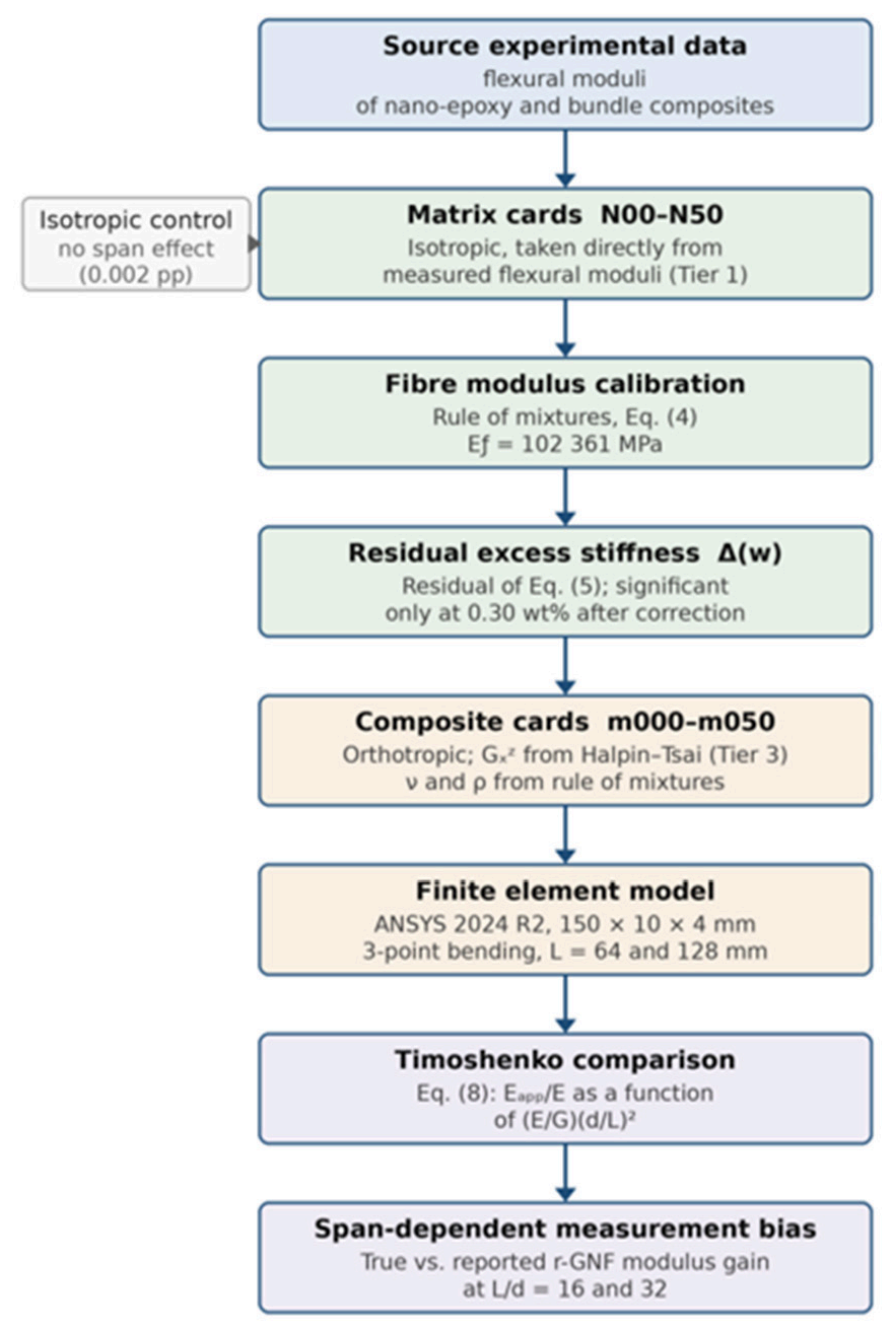
Workflow of the present study, from the source experimental data to the span-dependent measurement bias. Grey boxes indicate the verification steps.

The overall workflow is summarized in Figure 1. It proceeds in three stages. The isotropic matrix cards are taken directly from the measured flexural moduli of Refs. [6,7] and require no further assumption. The fibre modulus is then calibrated against the pure-epoxy bundle group, and the composite cards are assembled from these two sources together with the residual excess stiffness Equation (5). Each stage is verified independently before the finite element results are compared with the Timoshenko solution.

### 2.1. Source Data

Two published datasets form the basis of this study.

**Figure 2:**
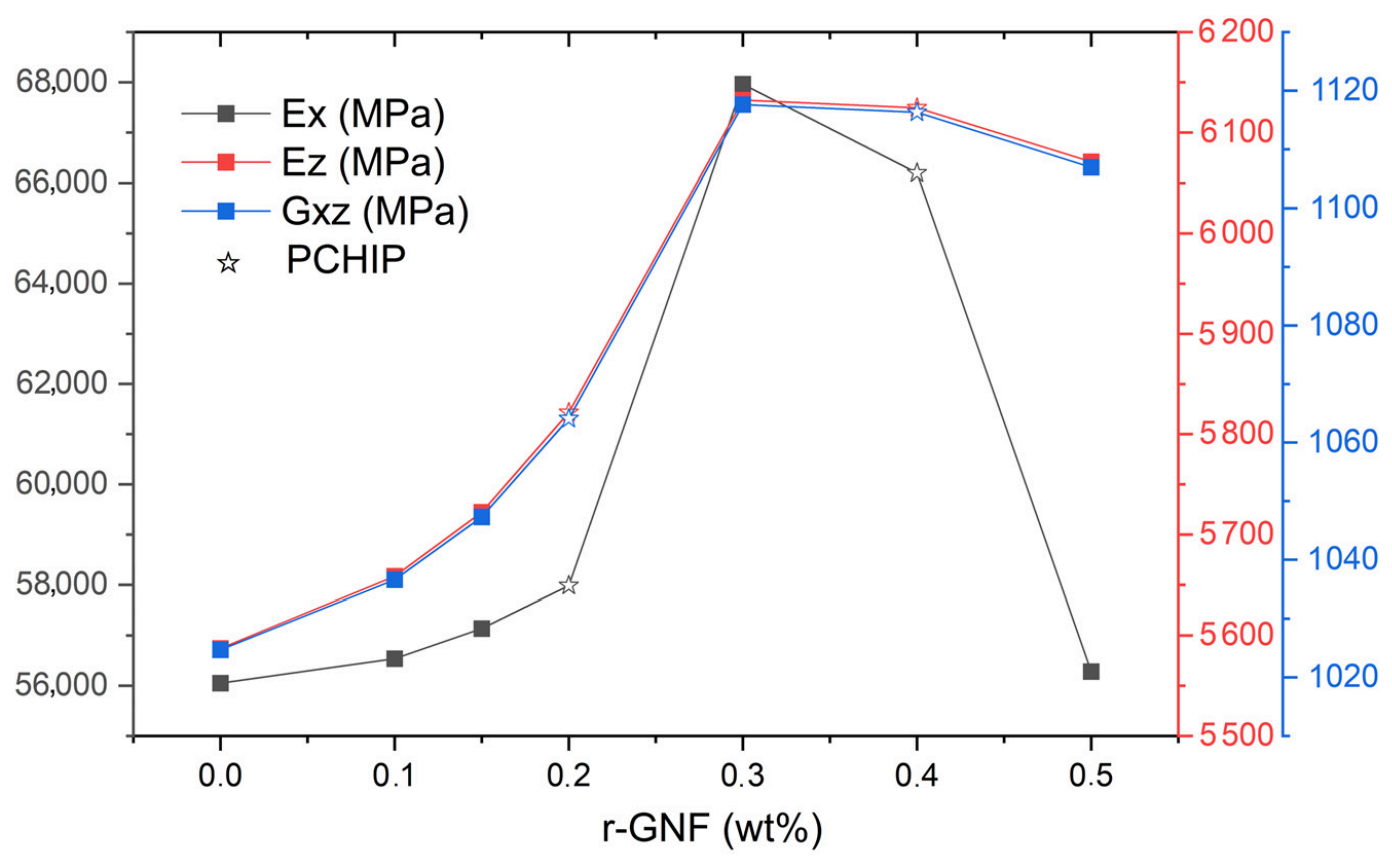
Evolution of the longitudinal (*E*_x_), transverse (*E*_y_), and in-plane shear (*G*_xz_) moduli as a function of r-GNF weight fraction, defining the orthotropic composite material cards. Note the distinct peak at 0.30 wt% driven by the residual excess stiffness.

The first is the flexural characterization of r-GNF-reinforced epoxy reported by Jana, Zhong, and Gan [6]. The resin was Epon 828 (Westlake Epoxy, Rotterdam, The Netherlands) cured with boron trifluoride-monomethylamine; r-GNFs were blended with butyl glycidyl ether diluent at a 1:6 ratio by weight and dispersed in the resin at loadings of 0.15, 0.20, 0.30, and 0.50 wt%. Three-point bending was performed at 1 mm/min in accordance with ASTM D790. Figure 2 of that work reports the initial modulus with its standard deviation for the neat resin and each nano-epoxy formulation; these five values constitute the entire matrix card library used here.

The second is the tensile study of UHMWPE fiber/nano-epoxy bundle composites by Jana, Hinderliter, and Zhong [7]. Bundles of 120 Spectra 1000 (Honeywell Intl. Inc., Charlotte, NC, USA) filaments were impregnated with pure epoxy or nano-epoxy and tested in tension. Also, Figure 2 of that work reports 40 individual specimen moduli together with the corresponding matrix volume fraction. These data are used here to calibrate the fiber modulus and to extract the r-GNF residual excess stiffness. Previous studies by Jana et al. [6] demonstrated that the addition of r-GNF significantly enhances the mechanical properties of the matrix. Specifically, increasing the r-GNF content from 0.00 wt% to an optimal 0.30 wt% resulted in a 20.5% increase in the elastic modulus (from 2770 to 3337 MPa) and a 25.9% improvement in flexural strength (from 132.2 to 166.4 MPa). Beyond 0.30 wt%, a slight degradation in properties was observed, likely due to agglomeration effects.

### 2.2. Material Card Construction

All material constants are classified into three tiers according to provenance. Tier 1 values are taken directly from measurement; Tier 2 values are derived from Tier 1 data using published relations; Tier 3 values are literature assumptions. This classification is retained throughout and is reported in full in the Supplementary Material.

#### 2.2.1. Matrix Cards

The five matrix cards are defined as isotropic. To accurately reflect the experimental behavior, the elastic moduli (*E*) for the varying r-GNF weight fractions (0.00 to 0.50 wt%) were parameterized directly matching the experimental values reported in Ref. [6], which range from a baseline of 2770 MPa up to a peak of 3337 MPa at 0.30 wt%. Poisson’s ratio (ν) was uniformly taken as 0.35 and density (ρ) as 1150 kg m^−3^ across all cards; neither parameter is reported in the original source, but both represent standard literature values for cured DGEBA epoxy. The neat-resin modulus of 2770 MPa is highly consistent with the manufacturer specifications for Epon 828, which states a modulus above 2750 MPa for unfilled systems. Furthermore, the shear modulus (*G*) for each configuration was not entered as an independent variable; rather, it was analytically derived within the model using the standard isotropic relation *G* = *E*/[2(1 + \ν)], yielding a constant *E*/*G* ratio of 2.700 for all matrix cards.

#### 2.2.2. Fiber Modulus Calibration

The rule of mixtures used in the source work [7,10] states:

*E_comp_* = *V_f_E_f_* + (1 − *V_f_*)*E_m_* (4)

where *E*_comp_ is the composite longitudinal modulus (MPa), *V*_f_ the fiber volume fraction (–), *E*_f_ the fiber modulus (MPa), and *E*_m_ the matrix modulus (MPa).

Applying this to the 10 pure-epoxy bundle specimens of Ref. [7] with the matrix modulus fixed at the measured value of 2770 MPa and fitting the fiber modulus by least squares yields *E*_f_ = 102,361 MPa. This falls within the manufacturer-quoted range of 93–135 GPa for Spectra 1000 fiber, providing an independent check on the calibration. The uncertainty in this calibration was estimated from the scatter of the same group. The mean residual of the 10 pure-epoxy specimens about the fitted line is +0.22 GPa with a 95% confidence interval of [−1.68, +2.13] GPa, corresponding to a standard error of 0.84 GPa on the mean. Dividing by the mean fiber volume fraction of that group, gives a standard error of 1.94 GPa on *E*_f_, or a 95% confidence interval of [98.0, 106.7] GPa. The relative uncertainty is therefore about 4%, and the interval lies entirely within the manufacturer-quıted range. Because *E*_f_ is calibrated on the unfilled group and the residuals of the remaining groups are referenced to it, this uncertainty propagates directly into Δ(w); it is already contained in the confidence intervals reported in Section 2.2.3. The fiber volume fraction for the card library was fixed at *V*_f_ = 0.535. The source dataset provides no single value common to all four treatment groups, as Section 3.5 documents, so any choice is necessarily a compromise. The value adopted here is the midpoint of the widest interval shared by three of the four groups (0.0, 0.2, and 0.3 wt%), which includes the two formulations on which the principal finding rests. The 0.5 wt% group lies entirely above it. Fixing *V*_f_ across library is deliberate: it removes fibre content as a variable so that the differences between cards reflect matrix modification alone, which is the quantity of interest. The consequence is that the cards represent a hypothetical series at constant fibre content rather than the specimens actually tested, and the effect of this idealization is examined in Section 5.5.

#### 2.2.3. Residual Excess Stiffness

**Figure 3:**
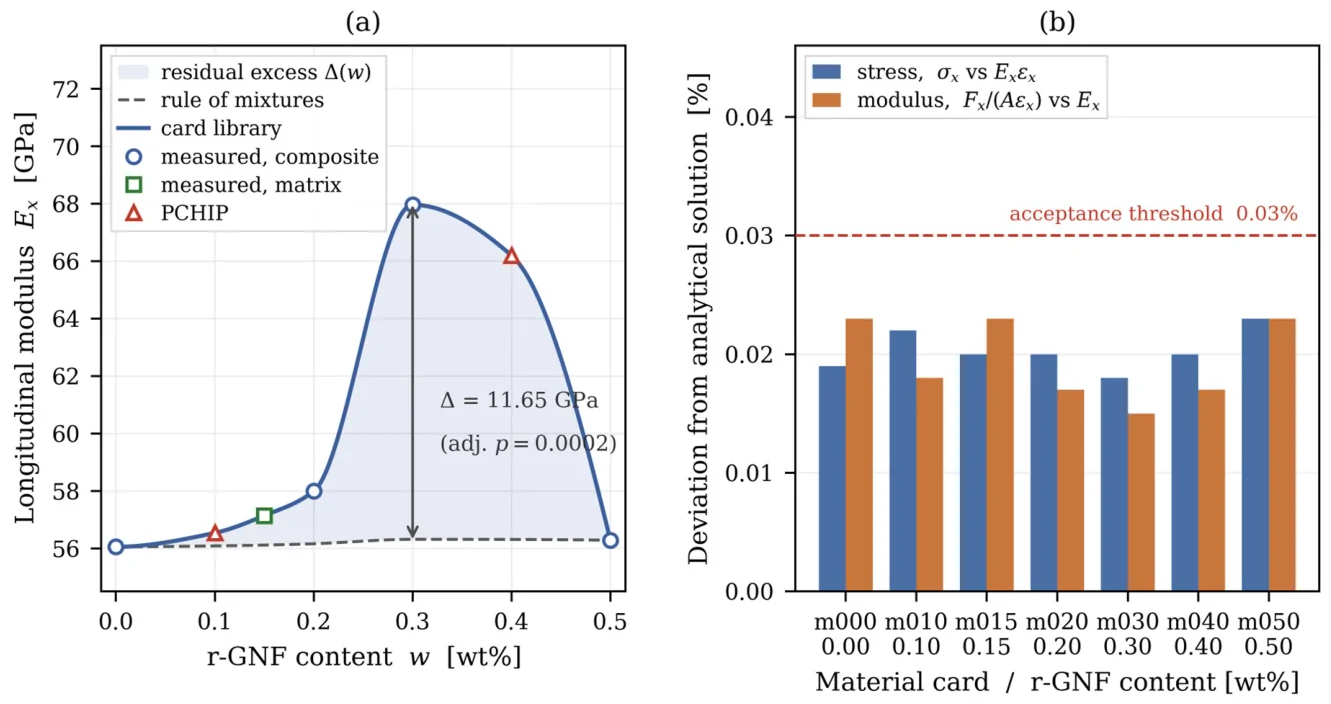
Card library construction and tensile verification. (**a**) Longitudinal modulus of the composite card library as a function of r-GNF content, showing the rule-of-mixtures baseline and the residual excess stiffness Δ(w) extracted in Section 2.2.3. (**b**) Deviation of the finite element result from the analytical solution for each card, on both the stress and the reaction-force route.

With E_f_ calibrated from the pure-epoxy group alone and E_m_(w) taken from Ref. [6], the residual of each treatment group with respect to the rule-of-mixtures prediction defines a residual excess stiffness, Δ(w). This term represents the excess stiffness not accounted for by the fiber and matrix contributions alone. To evaluate the significance of this excess, a one-sample *t*-test was applied to the residuals of the 40 bundle specimens derived from Ref. [7] (n = 10 per group) [11]. The analysis revealed a strong and highly significant effect at 0.30 wt% r-GNF (Δ(w) = +11.65 GPa,95% CI [+9.70, +13.60], raw *p* < 0.0001, adjusted *p* = 0.0002). At 0.20 wt%, the raw value reached nominal significance (Δ(w) = +1.83 GPa,95% CI [ +0.13, +3.53], raw *p* = 0.038) but did not survive correction (adjusted *p* = 0.114); the lower confidence bound lies close to zero, consistent with a marginal effect. The residuals for the 0.00 wt% and 0.50 wt% groups were +0.22 GPa (raw *p* = 0.797) and −1.15 GPa (raw *p* = 0.441), neither approaching significance before or after correction. Conversely, the residual excess stiffness for the 0.00 wt% and 0.50 wt% groups were +0.22 GPa (*p* = 0.797) and −1.15 GPa (*p* = 0.441), respectively. Because these latter values were not statistically distinguishable from zero, they were conservatively set to zero in the subsequent modeling phases. This decomposition is shown graphically in Figure 3a.

Because neither residual reached significance, both were set to zero in the card library. This is a modelling choice rather than a statistical necessity, and its consequence was checked directly. Retaining the point estimates instead of zeroing them raises *E*_x_ of the unfilled card by 0.39% and lowers *E*_x_ of the 0.50 wt% card by 2.04%, leaving the four intermediate cards unchanged. The true modulus gain at the optimum loading falls from 21.25% to 20.78%, a shift of less than half a percentage point, so the central finding of this work is insensitive to the choice. The 0.50% gain, however, changes sign, from +0.41% to −2.02%; this is consistent with the stoichiometric explanation offered in the source work, in which the higher diluent content required at that loading reduces the wpoxy cross-link density, but the underlying residual is not statistically distinguishable from zero and the negative value should not be read as a measured degradation. Zeroing was therefore retained as the conservative option.

#### 2.2.4. Composite Cards

The longitudinal modulus of each composite card follows from:

*E_x_*(*w*) = *V_f_E_f_* + *V_m_E_m_*(*w*)+Δ(*w*) (5)

where *E*_x_(*w*) is the composite longitudinal modulus at r-GNF weight fraction *w* (MPa), *V*_m_ = 1 − *V*_f_ is the matrix volume fraction (–), *E*_m_(*w*) is the measured matrix modulus at loading *w* (MPa), and Δ(*w*) is the residual excess stiffness (MPa).

The resulting orthotropic material properties for the seven composite configurations (ranging from 0.00 to 0.50 wt% r-GNF) are graphically summarized in Figure 2, defining the complete composite card library. Configurations m010 (0.10 wt%) and m040 (0.40 wt%) were generated via Piecewise Cubic Hermite Interpolating Polynomial (PCHIP) interpolation to ensure an even spacing across the filler content spectrum. For card m015 (0.15 wt%), the empirically measured matrix modulus at that loading was utilized, while the corresponding residual excess stiffness was interpolated. Across all seven composite cards, the volumetric rule of mixtures yielded a constant bulk density (ρ) of 1054 kg m^−3^. Similarly, the Poisson’s ratios derived from the rule of mixtures remained constant across all formulations, with the in-plane ratio (ν_xy_ = ν_xz_) evaluated at 0.302 and the out-of-plane ratio (ν_yz_) at 0.403.

The axial shear modulus of each card was obtained from the Halpin-Tsai semi-empirical relations [12]:

*G*_x_*z* = *Gm* (1 + *ξηVf*)/(1 − *ηVf*),   *η* = (*Gf*/*Gm* − 1)/(*Gf*/*Gm* + *ξ*) (6)

where *G*_m_ is the matrix shear modulus, *G*_f_ is the fibre axial shear modulus, ξ = 1 is for the axial shear, and ξ = 2 is for the transverse direction, following the standard values for circular fibres in a square packing array [10,12]. The fibre axial shear modulus is not reported for Spectra 1000; the convention of Chocron et al. for UHMWPE yarn was adopted [13], in which the axial shear modulus lies approximately two orders of magnitude below the axial tensile modulus, giving *G*_f_ = 1024 MPa.

Two assumptions are implicit in this route. The Halpin-Tsai relations assume uniformly distributed, perfectly aligned fibres of circular cross-section and a perfectly bonded interface, none of which is verified for the impregnated bundles of [7]. The Chocron convention was established for Dyneema HB80 (Avient Comp., Avon Lake, OH, USA), a UHMWPE grade of comparable molecular architecture but different processing history; its transfer to Spectra 1000 is an assumption rather than a measurement. Both quantities are Tier 3, and their influence on the results is examined in Section 5.2.

Consequently, the orthotropy ratio (*E*_x_/*G*_xz_) evolved continuously with r-GNF loading, peaking at a maximum anisotropy of 60.81 for the 0.30 wt% configuration (card m030), before relaxing back to 50.85 at 0.50 wt% (card m050). The provenance of each orthotropic constant should be kept in view when interpreting the finite element results that follow. The longitudinal modulus Ex is Tier 2: it combines the measured matrix moduli of [6] with the calibrated fibre modulus and the residual excess stiffness, the latter derived directly from the measured bundle data of [7]. The Poisson’s ratios and the density are also Tier 2, obtained by rule of mixtures from the same inputs. The transverse moduli *E*_y_ and *E*_z_ and all three shear moduli *G*_xy_, *G*_xz,_ and *G*_yz_ are Tier 3: they rest on the Halpin-Tsai relations combined with fibre properties that are assumed rather than measured for this system. The implications of this modeling convention are discussed extensively in Section 5.2.

The interpolation scheme was chosen deliberately. Because the response has a sharp maximum at 0.30 wt%, a cubic spline overshoots on either side of it and returns negative residuals at 0.10 and 0.15 wt%, which is physically meaningless; PCHIP is shape-preserving and does not. Comparing PCHIP with linear interpolation gives a measure of the remaining uncertainty: the gains of cards m010 and m015 shift by 0.84 and 0.62 percentage points, respectively, while card m040 is considerably more sensitive and shifts by 7.3 points. The interpolated cards are therefore reported as illustrative of the trend rather than as quantitative predictions. No conclusion of this work rests on them: the headline bias of 13.3% is the ratio between m030 and m000, both of which are built from measured matrix moduli and measured residuals.

### 2.3. Numerical Model

**Figure 4:**
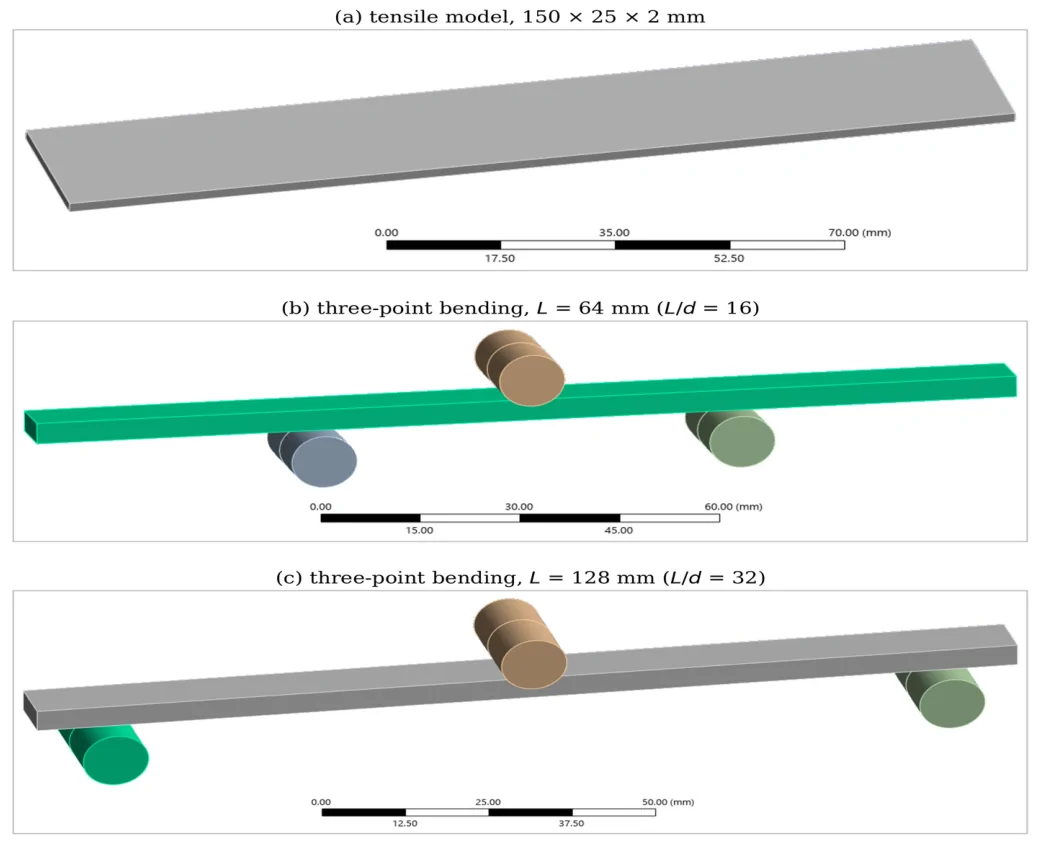
Finite element model geometry. (**a**) Tensile verification model, 150 × 25 × 2 mm, loaded by an axial displacement of 0.75 mm. (**b**) Three-point bending at L = 64 mm (L/d = 16) and (**c**) at L = 128 mm (L/d = 32), with the two lower supports fully fixed and a vertical displacement of 1.000 mm applied to the upper loading nose. The specimen is identical in (**b**,**c**), so span is the only variable between the two configurations.

All simulations were performed in ANSYS 2024 R2 Static Structural. The specimen was modelled as a 150 × 10 × 4 mm prismatic bar with the fiber direction aligned with the 150 mm axis, with the schematic description given in Figure 4. Three Ø10 mm, 20 mm-long steel cylinders were positioned tangent to the specimen: two lower supports and one upper loading nose. Tangency is exact in the assembly: the cylinder axes lie 5.000 mm from the specimen’s faces, so the surfaces are coincident to floating-point precision with no initial gap or interference. The two assemblies are provided as STEP files in the Supplementary Material. Two span configurations were used, L = 64 mm (L/d = 16, the ASTM D790 and ISO 178 default) and L = 128 mm (L/d = 32), on the same specimen, so that span is the only variable between the two configurations. The width-to-depth ratio of 2.5 keeps the specimen within beam behaviour and avoids the plate-like anticlastic constraint that arises at higher aspect ratios.

All three contacts were frictionless with augmented Lagrange formulation and asymmetric behaviour. The specimen was meshed at 1.0 mm, and the cylinders were meshed at 2.0 mm with quadratic solid elements, giving a fully structured hexahedral mesh of 150 × 10 × 4 elements in the specimen and 7000 elements with 35,848 nodes in the assembly as a whole. Four quadratic elements span the 4 mm depth, so nine nodes resolve the through-thickness stress distribution. The lower supports were fully fixed. A vertical displacement of 1.0 mm was applied to the loading nose in 20 equal substeps. Rigid-body translation along the specimen axis and rotation about the loading axis were suppressed using a deformable remote displacement applied to one side face, which constrains the mean motion of the face without introducing artificial stiffness. Large deflection was disabled and weak springs were not used.

Throughout this work, the deflection *D* entering the reduction formula *E*_app_ = *PL*^3^/(48*ID*) is the applied displacement of the loading nose, not the computed midspan deflection of the specimen. This choice mirrors the physical experiment, in which the reported deflection is crosshead travel; it is stated explicitly because the two quantities differ by a small offset discussed in Section 5.3.

Equilibrium was verified in every run by comparing the reaction at the loading nose with the summarized support reactions and by confirming that the in-plane reaction components were at machine-zero level. Across all 24 bending runs, the two reactions were equal and opposite in the loading direction to every reported digit, and the in-plane components did not exceed 10–13 N.

A separate tensile model was used to verify the orthotropic cards independently of the bending configuration: a 150 × 25 × 2 mm bar meshed at 0.5 mm with quadratic solid elements, giving a structured hexahedral mesh of 300 × 50 × 4 elements—60,000 elements and 289,909 nodes, with four elements through the thickness—with one end face fully fixed and an axial displacement of 0.75 mm applied to the other over the 150 mm gauge length, so that ε_x_ = 0.50%. This model contains no contact.

### 2.4. Verification and Validation Protocol

Three levels of checking were applied and are reported separately, since they establish different things. Implementation verification compares the finite element result with the closed-form solution for the same idealization. For tension, this is σ = *E*_x_ε; for bending, it is the Timoshenko solution [14]:

(7) $$P=\frac{D}{\frac{L^{3}}{48E_{x}I}+\frac{L}{4\kappa G_{\mathrm{xz}}A}}$$

where *G*_xz_ is the axial shear modulus (MPa), *A* = *bd* is the cross-sectional area (mm^2^), and κ is the shear correction factor (–), taken as 5/6 for a rectangular section [15].

This confirms that the material data have been entered correctly and the model solved correctly. Comparison with the source experiment places the back-calculated apparent modulus, *E*_app_ = *PL*^3^/(48*ID*), against the modulus measured in [6]. Because the matrix cards are taken directly from those measurements, this comparison verifies the implementation of the bending model and the reduction chain; it does not independently validate the material behaviour. Since the source moduli were themselves reduced using the Euler-Bernoulli relation, this is a like-for-like comparison. This is possible only for the matrix cards, since no experimental data exist for the plate geometry assumed for the composite. Consistency checking verifies that the ratio of results between cards reproduces the ratio of the input moduli, which detects entry errors independently of any absolute reference.

## 3. Results

### 3.1. Tensile Verification

All seven composite cards were run in uniaxial tension; results are given in Table 1. The measured longitudinal stress agreed with σ = *E*_x_ε to within 0.023% for every card, and the modulus back-calculated independently from the support reaction, *E*_x_ = *F*_x_/(*A*ε_x_), reproduced the input value to the same accuracy. Transverse reaction components were identically zero in every run, confirming the absence of spurious lateral restraint.

The deviations are uniformly positive, ranging from +0.018% to +0.023% on stress and from +0.015% to +0.023% on the reaction-derived modulus, with a scatter about the mean of +0.020% comparable to the five-significant-figure precision of the solver output. The mesh study of Table 2 shows that this residual is not a discretization error: it is unchanged to four decimal places over a 64-fold increase in element count. Quadratic shape functions represent the linear displacement field of uniaxial tension exactly, so the gauge section carries no discretization error at any of the mesh sizes examined. The residual therefore originates in the boundary condition rather than the solution: the fixed end face suppresses lateral contraction and stiffens the specimen slightly, which raises the reaction above the free-uniaxial value. This is the same end effect that governs the Poisson results below, and it is consistent with the deviation being uniformly positive. The magnitudes are two orders below the 0.5–5% experimental scatter of the source data and are of no practical consequence, but they are reported in full because identifying their origin determines which quantities may be read from the model.

Poisson’s ratio was checked independently. The through-thickness strain gave ν_xz_ = 0.3035 against the card value of 0.302 (+0.50%). The width-direction strain gave ν*xy* = 0.2962 (−1.93%); this larger deviation is attributable to the Saint-Venant end zone, where the fixed face suppresses lateral contraction over a length comparable to the cross-sectional dimension. Since that dimension is 25 mm in the width direction but only 2 mm through the thickness, the whole-body average is depressed in the former and essentially unaffected in the latter. Two features of the data confirm that this is a geometric reporting artefact rather than a card error. First, the extremum of the width-direction strain field falls to −6 × 10^−8^, i.e., contraction is completely suppressed at the fixed face, whereas the corresponding through-thickness extremum remains at −1.3 × 10^−4^. Second, the deviation is constant to four decimal places across all seven cards, whose stiffnesses differ by up to 21%; a card error would vary between them. The practical implication is that Poisson’s ratio extracted from a full-body average will be low by roughly 2% in the wide direction.

A mesh convergence study was carried out on the tensile model of m000, the orthotropic composite card at 0 wt%, at four element sizes from 1.00 mm to 0.25 mm, spanning 7500 to 480,000 elements and 0.13 to 6.35 million degrees of freedom (Table 2). Node counts match the closed-form expression for a structured mesh of 20-node hexahedra at every size, confirming that the mesh is fully structured throughout. The reaction force is identical to all reported digits across the four meshes and the volume-averaged stress varies by 0.005%, so every quantity used in Table 1 is mesh-independent.

The peak stress behaves differently. It rises monotonically by 19.0% between the coarsest and finest mesh and does not converge; a power-law fit gives σ_max_ ∝ *h^−^*^0.126^ with *R*^2^ = 0.9959. This is the signature of a stress singularity at the edge where the fixed face meets the free lateral surfaces, where the suppression of lateral contraction is discontinuous. The peak value is therefore a property of the mesh rather than of the material and must not be reported as a stress; the volume average used throughout this work is the appropriate quantity, and it is unaffected. The same caution applies to the compressive extremum beneath the loading nose in the bending model, discussed in Section 3.3.

The 0.50 mm mesh was adopted for the production tensile runs. The bending model uses 1.0 mm in the specimen, giving four quadratic elements and nine nodes through the depth.

### 3.2. Matrix Three-Point Bending: Comparison with the Source Experiment

**Figure 5:**
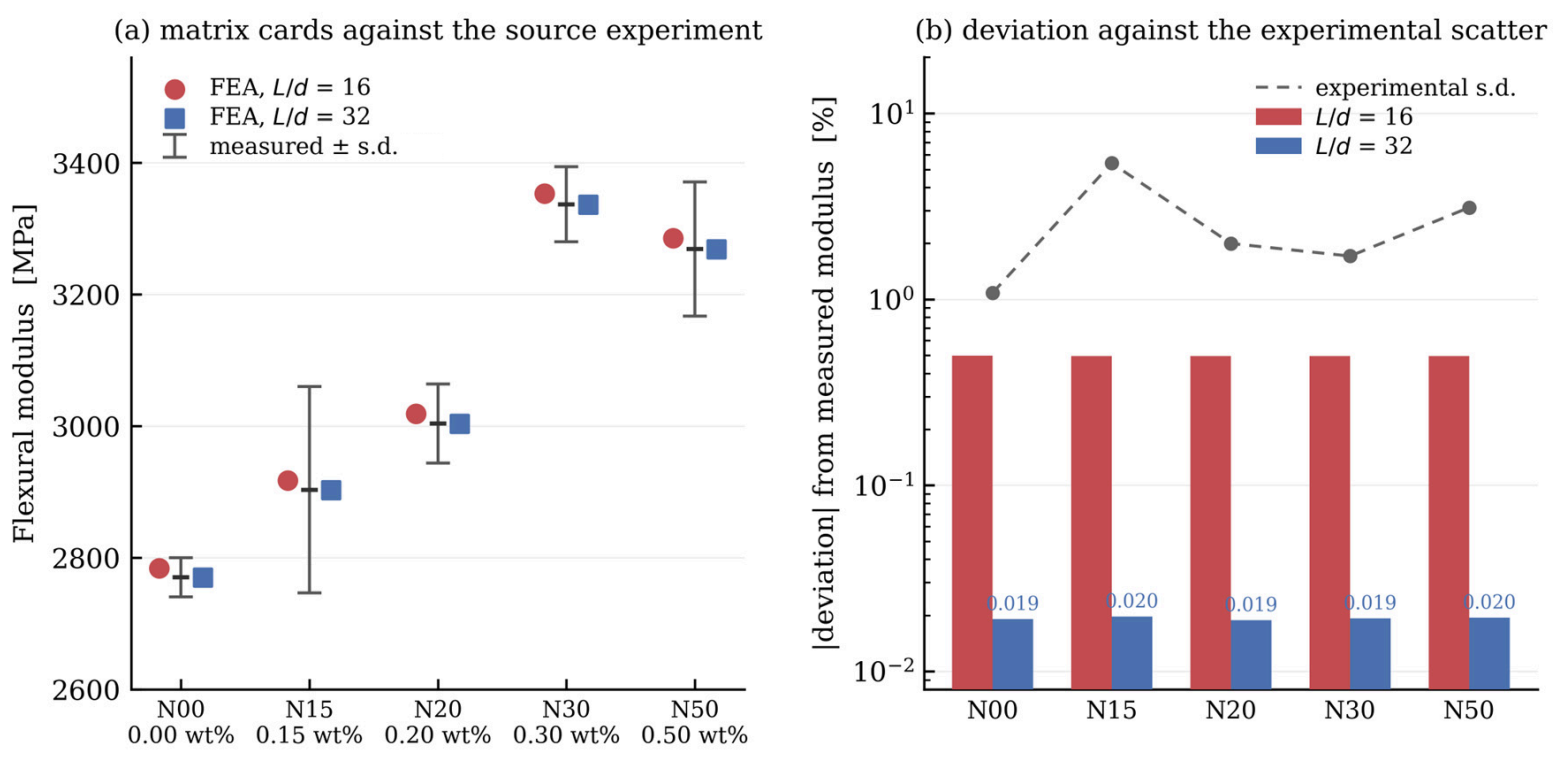
Matrix cards against the source experiment. (**a**) Apparent modulus at both spans compared with the measured modulus and its experimental standard deviation [6]. (**b**) Absolute deviation on a logarithmic scale, against the experimental scatter; a logarithmic axis is required because the two spans differ by more than an order of magnitude in accuracy.

The five isotropic matrix cards were run at both spans. Results are given in Table 3. At *L*/*d* = 16, the back-calculated apparent modulus exceeded the measured value by 0.494–0.496% for every card; at *L*/*d* = 32, the deviation fell to −0.019 to −0.020%. In all 10 runs, the apparent modulus lies inside the experimental standard deviation reported in Ref. [6], with a margin of between one and two orders of magnitude (Figure 5b).

The error budget at *L*/*d* = 16 is instructive. Transverse shear reduces the apparent modulus by 1.25%, a figure recovered exactly by the Timoshenko solution for *E*/*G* = 2.70 at this span ratio. Working against this, the contact point on the cylindrical supports migrates inward as the specimen rotates, shortening the effective span; for a support rotation of 0.0463 rad on a Ø10 mm cylinder, this amounts to 0.23 mm per support, or a 0.72% reduction in span, which raises the apparent stiffness by approximately 1.8%. The finite element reaction exceeds the Timoshenko prediction by 1.768% at this span, in close agreement with that estimate. The two effects very nearly cancel, leaving a net error of +0.50%. At *L*/*d* = 32, the support rotation halves, and the contact-shift term falls to +0.297%, while the shear term falls to 0.315%, giving a net error of −0.02%.

### 3.3. Composite Three-Point Bending

The seven orthotropic cards were run at both spans with the identical specimen geometry, contact definitions, mesh and boundary conditions used for the matrix. Agreement with the Timoshenko solution was within 0.263% at *L*/*d* = 16 and 0.161% at *L*/*d* = 32.

The apparent modulus behaves entirely differently from the matrix case. At *L*/*d* = 16, the shear contribution to midspan deflection is 19.2–22.2%, and the apparent modulus is only 77.6–80.7% of the true longitudinal modulus. At *L*/*d* = 32, the shear contribution falls to 5.6–6.7%, and the apparent modulus rises to 93.3–94.3% of the true value.

The peak longitudinal stress follows the same qualitative pattern, exceeding the elementary beam value 3*PL*/(2*bd*^2^) by far more in the composite than in the matrix at the same span, and by less at the longer span. That behaviour is again a consequence of shear deformation, which redistributes the stress field away from the linear through-thickness distribution assumed by the elementary relation. The magnitudes are not reported here because they are not mesh-converged, as Table 4 documents; the modulus results, which rest on reaction forces rather than on local stresses, are unaffected.

The comparison with the matrix control is summarized in Table 5. Since geometry, span, contact treatment, mesh, and loading are identical between the two material classes, the difference is attributable solely to elastic anisotropy.

A mesh study of the bending configuration was carried out on card m030 at *L* = 64 mm, the case with the highest anisotropy ratio and the largest stress deviation, at element sizes of 2.0, 1.0, 0.75, and 0.5 mm (Table 4). The reaction force, from which every modulus reported in this work is obtained, is essentially resolution-independent: it varies by 0.55% across a four-fold change in element size, and by 0.008% between the 1.0 and 0.75 mm meshes. The apparent modulus follows it directly, ranging from 52 863 to 52 572 MPa over the same interval. The production mesh of 1.0 mm therefore returns a modulus converged to better than half a percent, which is two orders below the effect the paper reports.

The two stress extrema behave differently from each other and from the reaction. The compressive extremum beneath the loading nose does not converge: successive refinements change it by 62, 35, and 41 MPa, increments that do not decay, which is the contact-edge singularity already identified in the tensile study of Section 3.1.

The tensile-face maximum was examined more closely because a maximum taken over the whole body could in principle be reporting a contact concentration near a support rather than the bending stress at midspan. A path was therefore defined across the width of the lower face at midspan, where no contact occurs, and the stress along it was recorded at each mesh. The path values are given in Table 4. Two things follow. First, the whole-body maximum coincides exactly with the path value at the free edge at every mesh, so the reported maximum is the midspan tensile stress and not a contact artefact; the concern that motivated the check does not apply here. Second, the stress is almost uniform across the width, the centre, and the free edge, differing by 0.08% to 0.32%.

The tensile-face stress nevertheless converges slowly. A power-law fit to the path centre gives σ_x_ = 360.7 − 27.9·h1.22 MPa, placing the production mesh 6.5% below the extrapolated limit and the finest mesh 2.8% below it. The limit itself lies 16.7% above the elementary beam value, so the departure from beam theory is real and large, but its magnitude cannot be quoted from these meshes. Peak stresses are therefore not reported as results anywhere in this work. The modulus quantities, which rest on the reaction, are unaffected. All entries derive from reaction forces, which the mesh study of Table 4 shows to be the resolution-independent quantity; peak stresses are not tabulated here for that reason.

#### Stress Field Analysis and Transverse Shear

**Figure 6:**
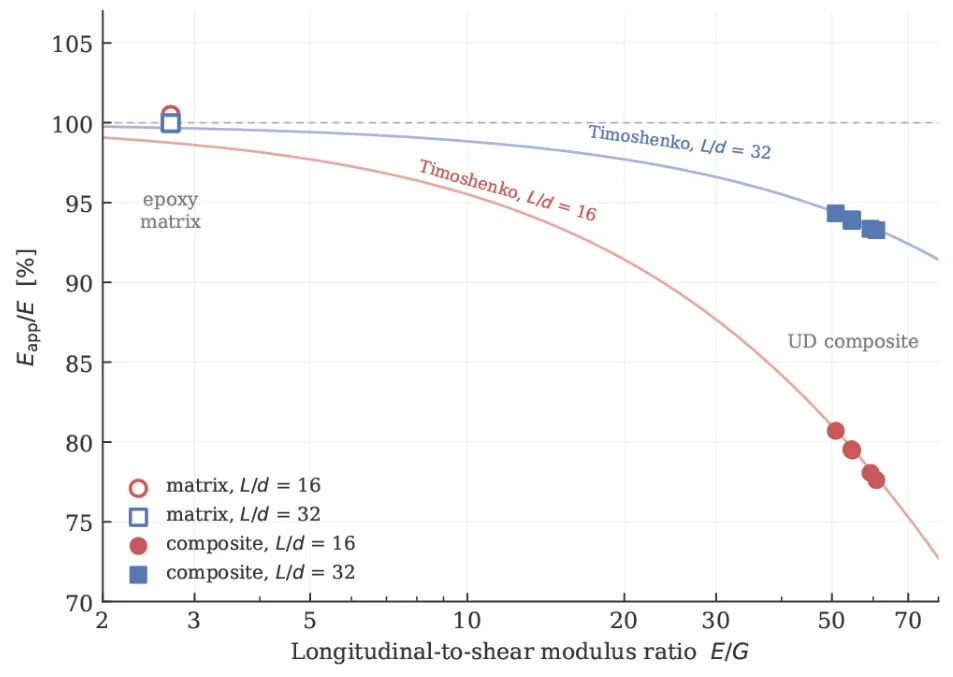
Apparent modulus ratio as a function of the longitudinal-to-shear modulus ratio, for both material classes at both spans, with the Timoshenko prediction *E*_app_/*E* = [1 + κ − 1(*E/G*)(*d*/*L*)^2^] − 1 shown as continuous curves. The composite points lie on the analytical curves; the matrix points lie slightly above them because the contact-shift term, which the analytical solution does not contain, exceeds the shear term at this anisotropy ratio.

**Figure 7:**
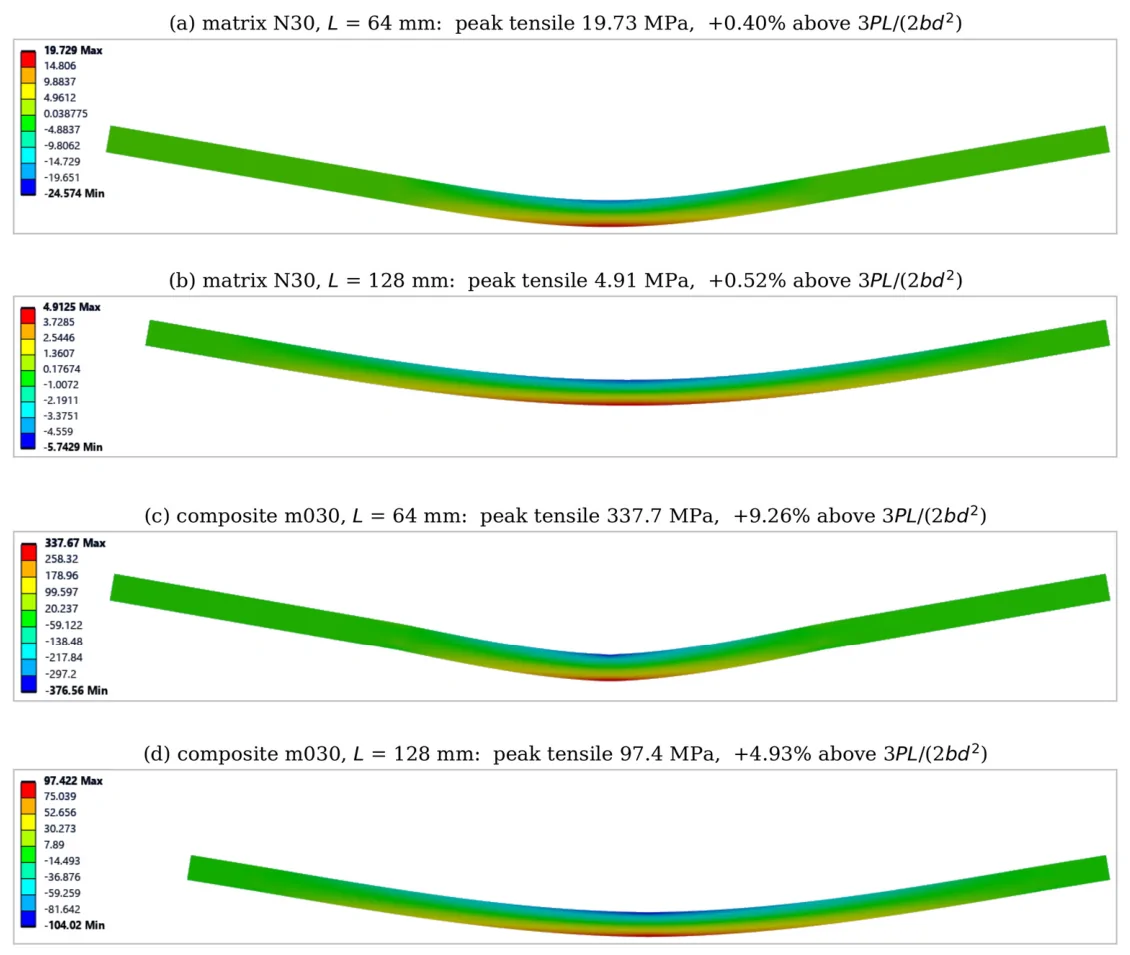
Longitudinal stress field on the deformed shape for the 0.30 wt% formulation at both material classes and spans. (**a**) Isotropic matrix card N30 at *L* = 64 mm; (**b**) N30 at *L* = 128 mm; (**c**) orthotropic composite card m030 at L = 64 mm; (**d**) m030 at *L* = 128 mm. All views share the same magnification. Deformation is shown at an automatic scale (true midspan deflection is 1.000 mm on a 150 mm specimen). Note that color scales are independent for each panel.

As can be seen on the Figure 6 the longitudinal-to-shear modulus ratio can be observed. To further investigate it, Figure 7 compares the longitudinal stress fields for the 0.30 wt% formulation across different material classes and spans. Reading down each pair isolates the effect of the span at a constant material, while comparing the matrix (Figure 7a,b) with the composite (Figure 7c,d) isolates the effect of anisotropy at a constant span. The peak tensile stress on the lower face exceeds the elementary beam value, 3*PL*/(2*bd*^2^), by 0.40%, 0.52%, 9.26%, and 4.93%, respectively. Notably, doubling the span leaves the isotropic matrix essentially unchanged but halves the excess stress in the orthotropic composite, which is a definitive signature of transverse shear. When interpreting these stress fields, two specific features require care:

*Independent Color Scales:* Under a prescribed displacement, the reaction differs by a factor of 17 between the matrix and the composite. Utilizing a common color scale would render the matrix panels visually uniform and uninformative. Therefore, the panels should be compared based on the shape of the through-thickness stress distribution rather than by absolute color mapping.

*Contact Stress Superposition:* The compressive extremum on the upper face exceeds the tensile peak in magnitude in all four cases: by 24.6%, 16.9%, 11.5%, and 6.8%, respectively. This deviation is caused by the local contact stress beneath the loading nose superposed on the bending field, rather than a true bending asymmetry. Because this effect is largest in the matrix (where the underlying bending stress is the smallest), the deviations quoted in Table 5 are computed exclusively from the tensile face.

### 3.4. Formulation Dependence of the Measurement Bias

The apparent-modulus loss is not constant across the card library. It correlates with the anisotropy ratio *E*_x_/*G*_xz_, which varies from 50.8 for the 0.5 wt% card to 60.8 for the 0.3 wt% card. The reason is that r-GNFs do not scale the longitudinal and shear moduli equally: at the optimum loading, the longitudinal modulus rises by 21.2%, while the axial shear modulus rises by only 9.1%, so the anisotropy ratio increases and the shear penalty grows.

The consequence for the reported filler performance is direct. Table 6 compares the true modulus gain, defined by the ratio of longitudinal moduli, with the gain that a standard flexural test would report at each span. At the optimum loading of 0.3 wt%, a 16:1 span reports +18.42% against a true value of +21.25%, an underestimate of 2.83 percentage points, or 13.3% of the gain. At 32:1, the reported value rises to +20.41%, an underestimate of 0.84 points or 3.9%.

The distortion is largest for the two high-performing formulations, but it is not confined to them. Below 0.2 wt%, the reported and true gains agree to within 0.1 percentage point because the anisotropy ratio barely changes over that interval. The bias therefore does not act as a uniform correction factor across the composition series. It is sign and magnitude that follows the movement of the anisotropy ratio relative to the reference card rather than the size of true gain. Where the ratio rises, as at 0.30 and 0.40 wt%, the gain is, below, reported; where it falls, as at 0.50 wt%, the gain is over-reported, and the reported value of 1.97% exceeds the true value of 0.41% by nearly a factor of five. A single correction factor applied across a composition series would therefore be wrong in both directions.

### 3.5. Observations on the Source Bundle-Composite Dataset

Two issues in the dataset of Ref. [7] were identified during card construction and are reported here because they bear on how that dataset should be interpreted.

**Figure 8:**
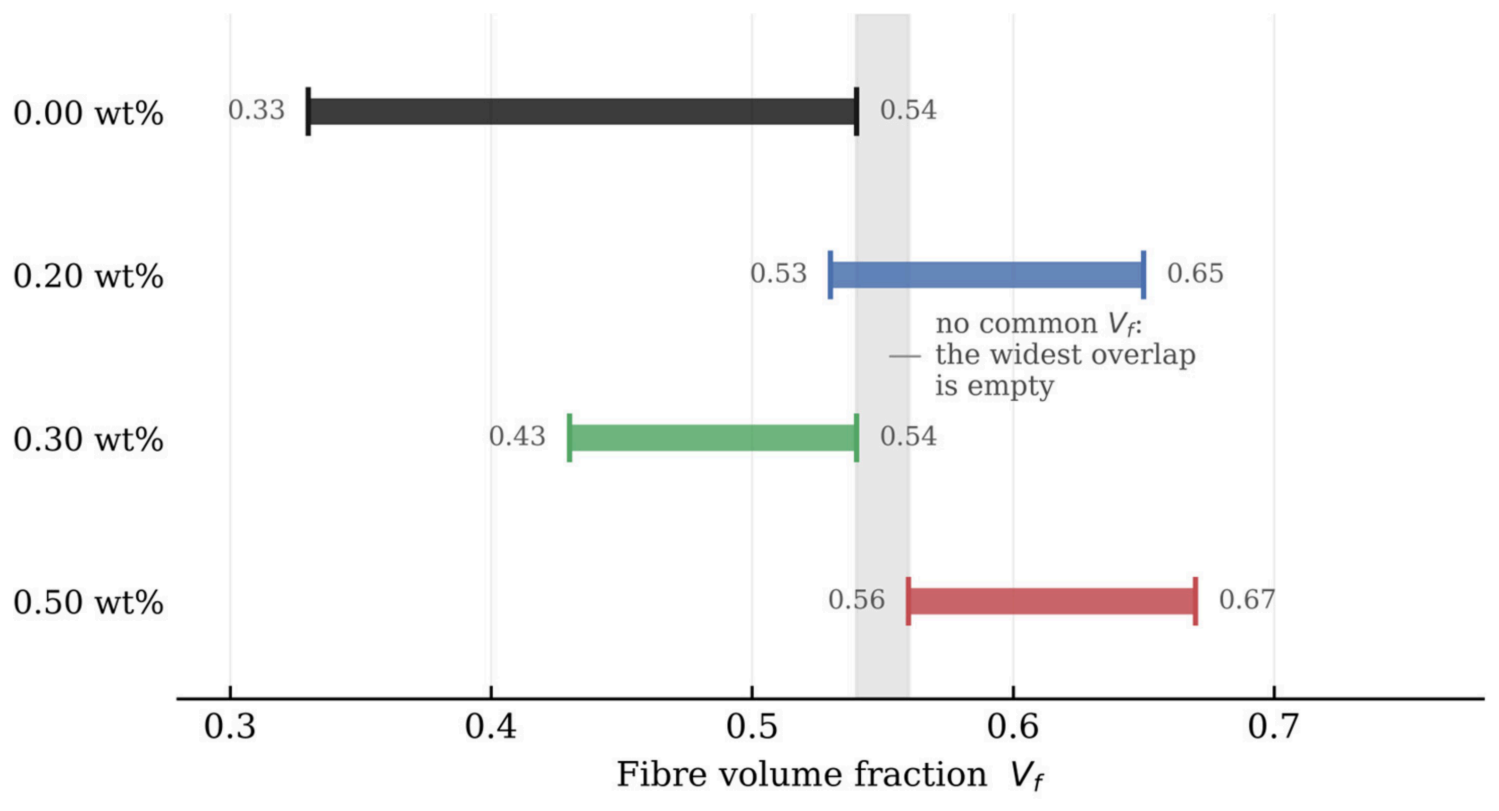
Fiber volume fraction range of each treatment group in Ref. [7], n = 10 per group. The shaded band marks the gap between the highest group minimum and the lowest group maximum; no value of *V*_f_ is common to all four groups.

First, the four treatment groups do not share a common fiber volume fraction (Figure 8). The pure-epoxy group spans *V*_f_ = 0.33–0.54 and the 0.5 wt% group spans *V*_f_ = 0.56–0.67; the highest group minimum, 0.56, exceeds the lowest group maximum, 0.54, so the intersection across all four groups is empty. Since the longitudinal modulus of a fiber composite is dominated by *V*_f_, with a sensitivity of approximately −75 to −87 GPa per unit change in matrix volume fraction in this dataset, direct comparison of raw group moduli conflates the filler effect with the fiber content. The raw group means illustrate the problem: 46.8 GPa at 0.0 wt% against 63.3, 63.6, and 62.9 GPa at 0.2, 0.3, and 0.5 wt%, an apparent step that largely tracks the shift in *V*_f_ rather than the filler. The rule-of-mixtures decomposition applied in Section 2.2.3 removes this confound and shows that only the 0.3 wt% group exhibits a genuine excess above the fiber contribution.

**Figure 9:**
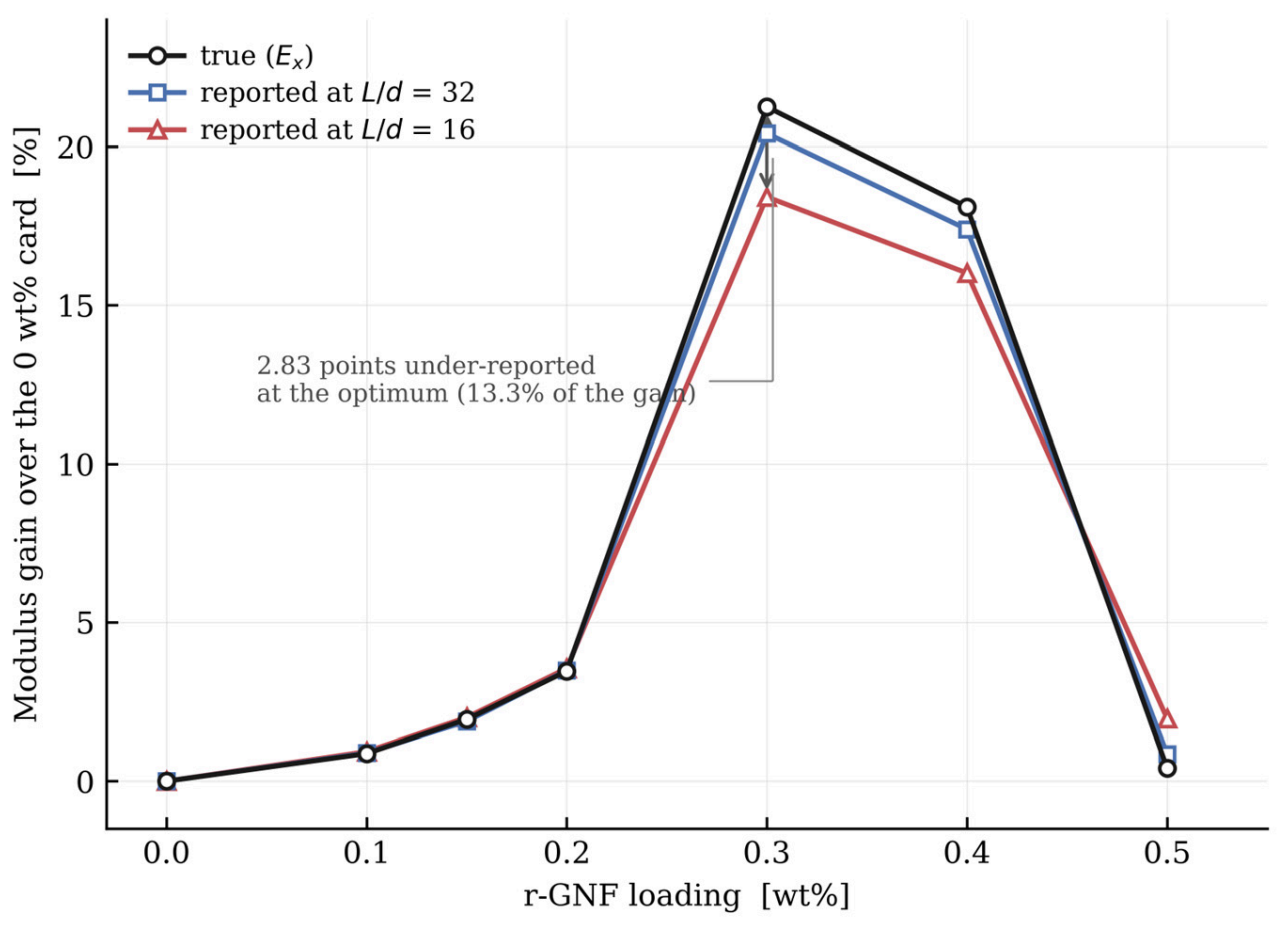
True modulus gain compared with the gain a standard flexural test would report at each span ratio. The three curves are indistinguishable below 0.2 wt%, and separate only where the anisotropy ratio rises.

Second, the empirical model of Ref. [7] is reported to attain its maximum of 66.28 GPa at a matrix volume fraction of 38.5% and an r-GNF content of 0.33%. This number is chosen in accordance with the Figure 9. Numerical optimization of the published coefficients reproduces the maximum value of 66.27 GPa but locates it at a matrix volume fraction of 0.329 and an r-GNF content of 0.385%. The two coordinates appear to have been transposed in the text; the magnitude is unaffected.

## 4. Discussion

### 4.1. Mechanism

The results follow directly from the relative weight of the shear term in the Timoshenko deflection expression. The ratio of shear to bending compliance scales as:

(8) $$\frac{C_{s}}{C_{b}}=\frac{1}{\kappa }\frac{E}{G}{\left(\frac{d}{L}\right)}^{2}$$

so that the apparent modulus reduces to:

(9) $$\frac{E_{\mathrm{app}}}{E}={\left[1+\frac{1}{\kappa }\frac{E}{G}{\left(\frac{d}{L}\right)}^{2}\right]}^{-1}$$

where *C*_s_ and *C*_b_ are the shear and bending contributions to the midspan compliance (mm N^−1^), *E*_app_ is the apparent flexural modulus obtained from Equation (3), and *E*/*G* is the ratio of longitudinal to shear modulus (–).

The shear penalty is therefore proportional to *E*/*G*. For the epoxy matrix, *E*/*G* = 2.70 and the penalty at *L*/*d* = 16 is 1.25%. For the unidirectional composite, *E*_x_/*G*_xz_ ≈ 55 and the penalty is 20 times larger. Doubling the span ratio reduces the penalty by a factor of four, which is why the 32:1 configuration recovers most of the true modulus. Figure 6 shows that the composite results lie on this curve for both spans, so the finite element model reproduces shear-deformable beam theory without adjustment; the matrix results lie slightly above it, by the contact-shift term that the analytical solution does not contain.

The formulation dependence arises because the two moduli entering this ratio have different micromechanical origins. The longitudinal modulus is fiber-dominated and increases with filler content only through the matrix term and the interfacial residual excess stiffness; the axial shear modulus is matrix-dominated throughout. The measured gains of 21.2% in *E*_x_ and 9.1% in *G*_xz_ at the optimum loading therefore push the anisotropy ratio upward, and the measurement bias with it.

### 4.2. Implications for Flexural Testing of Anisotropic Composites

The default 16:1 span ratio of ASTM D790 and ISO 178 is well suited to isotropic polymers, and the matrix control runs in this study confirm that: the standard configuration returned the measured modulus to within 0.5%, with the shear deficit almost exactly offset by the contact-point migration on cylindrical supports. For a unidirectional composite with the same geometry. However, the same configuration underestimated the longitudinal modulus by roughly 20%.

The existence of this effect is not new. Jalali and Taheri exploited it directly, using tests at two or more span ratios to extract the longitudinal and shear moduli simultaneously [16], and Mujika, examining five spans on unidirectional carbon/epoxy, noted that the longitudinal-to-shear modulus ratio exceeds 20 for such materials against a maximum of 3 for isotropic solids [17]. Errors of up to 18% in apparent flexural rigidity at short spans have been reported for unidirectional CFRP over a slenderness range of 10 to 65 and recovered by Timoshenko theory [18], and recent work concludes that conventional flexural testing is unsuitable for highly anisotropic materials [19]. The standards themselves permit longer spans for high-modulus materials [5].

In much of that work, the material is held fixed: the span is varied, and a correction or identification procedure follows. Less attention appears to have been given to how the bias behaves when the material itself changes across a composition series. In the present case, it does not appear to act as a constant. Because r-GNFs raise the longitudinal modulus considerably more than the axial shear modulus, the anisotropy ratio becomes a function of filler content. Table 6 illustrates the consequences: at 0.30 and 0.40 wt%, the gain is under-reported by 2.83 and 2.09 percentage points, while at 0.50 wt%, it is over-reported, with the predicted 1.97% exceeding the true 0.41% by nearly a factor of five. The error therefore appears to change sign within a single composition series, which suggests that a single multiplicative correction would not be adequate.

This points to a formulation-specific correction rather than a revision of the default span ratio: a standard that prescribes a single ratio cannot remove a bias whose sign depends on the material being tested. A practical recommendation follows, with the scope of the present model in mind. For the UHMWPE/r-GNF system examined here, over the anisotropy range 50.8 ≤ *E*_x_/*G*_xz_ ≤ 60.8, either a span ratio of at least 32:1 should be used, or the reported modulus should be corrected using the Timoshenko relation with an independently determined shear modulus. The same reasoning applies in principle to any unidirectional system with a comparably high longitudinal-to-shear modulus ratio, but the quantitative thresholds reported here were not tested outside that range and should not be transferred without verification.

### 4.3. Implications for the Reported r-GNF Performance

The finding that only the 0.3 wt% loading exhibits a statistically significant residual excess stiffness supports the central conclusion of Ref. [7] while refining its mechanism. The apparent monotonic improvement suggested by the raw group means is substantially an artefact of the differing fiber volume fractions between groups; once this is removed, the 0.2 wt% effect is marginal and the 0.5 wt% effect vanishes. This is consistent with the stoichiometric explanation offered in the source work, in which the higher diluent content required at 0.5 wt% reduces the epoxy cross-link density and offsets the nanofiber contribution [6,7].

It should be stressed that this concerns modulus only. The flexural and tensile strength data of Refs. [6,7] show improvement at all loadings, and the strength mechanism—crack deflection and improved interfacial adhesion—is distinct from the load-transfer mechanism that governs stiffness. The present analysis does not address strength.

## 5. Limitations

This is a computational reanalysis and simulation study; no new experimental measurements were conducted. Several limitations follow and should be carefully weighed when interpreting the results.

### 5.1. Lack of Direct Composite Experimental Validation

While the isotropic matrix model (N00–N50) was rigorously validated against source experimental data, the orthotropic composite models rely on derived material properties. Consequently, the composite bending results lack direct experimental validation within this study. The findings represent a theoretical limit of what standard beam theory predicts for these formulations, rather than empirical test outcomes.

### 5.2. Sensitivity to the Shear Modulus Assumption

The magnitude of the measurement bias is fundamentally governed by the anisotropy ratio (*E*_x_/*G*_xz_). Because direct experimental values for the axial shear modulus of UHMWPE yarn are notoriously difficult to obtain, *G*_xz_ was derived using the accepted Chocron approximation rather than empirical measurement. This imparts a degree of quantitative uncertainty to the exact percentage of modulus loss. However, as demonstrated by the parametric sweep in Table 7, the qualitative conclusion, that a standard 16:1 span significantly underreports the modulus gain at optimum loadings, remains robust across an 11-fold variation in the assumed shear modulus.

Quantitatively, an 11-fold variation in the assumed shear modulus moves the under-reported gain at the optimum loading between 6.5% and 15.0% of the true gain. The baseline row of Table 7 gives 12.7%, evaluated from the analytical Timoshenko solution; the corresponding finite element value for the same scenario is 2.83 points, or 13.3% of the gain, as reported in Table 6. The difference between the two lies within the finite element to analytical agreement range given in Table 5, and the sign and order of magnitude of the bias are therefore set by the anisotropy of the system rather than by the shear modulus assumption. A direct measurement would refine this figure without altering the conclusion. Two established routes are available: off-axis tensile testing at ±45°, from which the axial shear modulus follows through the transformed compliance relations, and the Iosipescu shear method of ASTM D5379 [20]. Inverse identification from the impact or modal response offers a third route [21,22]. Any of these would replace the present Tier 3 assumption with a Tier 1 value and is recommended before the correction proposed here is applied quantitatively to a specific formulation.

### 5.3. Contact Detection Artifact

A systematic offset (*D*_0_ = 9.37 μm) was identified in the load-displacement response of the bending models. This is a contact-detection artifact originating from the faceted meshing (sagitta effect) of the cylindrical target surfaces. It can be natively suppressed in future analyses by enabling solver-level “contact geometry correction.” Because this constant offset cancels out exactly when calculating the relative modulus ratios between formulations, it does not compromise the core comparative findings of the study. The matrix validation of Section 3.2 is itself an absolute comparison against experiment that the offset would have to pass through, placing an empirical bound on how much it distorts the absolute moduli.

### 5.4. Mesh Convergence and Local Stresses

Mesh convergence has been established for reaction forces, ensuring that every modulus reported here carries resolution independence. Local stresses, however, do not. The compressive extremum is singular in both configurations. The tensile-face maximum converges slowly and remains 6.5% below its extrapolated limit on the production mesh. Peak stresses are consequently not reported as results in this work, as no conclusion herein depends on them.

### 5.5. Hypothetical Specimen Geometry

The 150 × 10 × 4 mm test plate used in the finite element models is a hypothetical geometry. The original source data [7] were derived from micro-bundle specimens measuring 0.37–0.45 mm in diameter. Consequently, the simulations reflect how a standard macroscopic specimen would behave under ASTM/ISO guidelines, rather than serving as a direct geometric replication of the source tests.

The consequence of this idealization is limited for the quantity reported here. The apparent-to-true modulus ratio of Equation (9) depends only on the anisotropy ratio and on (*d/L*)^2^, not on the absolute specimen dimensions, so a change of scale alone does not alter the predicted bias. What the idealization does affect is the fibre volume fraction and the fibre arrangement: the model assumes a single value of *V*_f_ = 0.535 and a perfectly aligned, uniformly distributed reinforcement, whereas the source bundles span *V*_f_ = 0.33 to 0.67 with the packing irregularity characteristic of impregnated filament bundles. Both would modify *E*_x_ and *G*_xz_, and therefore the anisotropy ratio, although the sensitivity study of Section 5.2 indicates that the direction of the bias is preserved across a wide range of that ratio.

### 5.6. Model Exclusions (Damage Mechanics and Transverse Properties)

The analysis is strictly limited to linear-elastic, small-deflection regimes. Failure, damage, and the longitudinal splitting characteristic of unidirectional composites in bending are deliberately outside its scope, and no claims regarding strength are made. Additionally, the transverse modulus and transverse shear modulus are not exercised by either the tensile or the bending configuration and therefore remain unvalidated. Finally, the fixed fiber volume fraction of 0.535, justified in Section 2.2.2, does not represent the 0.5 wt% group, which lies entirely outside it.

## 6. Conclusions

A traceable orthotropic material card library for UHMWPE fiber/r-GNF nano-epoxy composites was constructed from two published datasets and evaluated in three-point bending by finite element analysis, with the isotropic nano-epoxy matrix serving as a reference case for which measured flexural moduli exist. The principal findings are:

Numerical findings.

- Matrix cards taken directly from measured flexural moduli reproduced the source experimental values in simulation with a maximum error of 0.50% at a 16:1 span ratio and 0.020% at 32:1; all 10 runs fell within the reported experimental standard deviation.
- For the unidirectional composite, with *E*_x_/*G*_xz_ between 50.8 and 60.8, the same specimen geometry and the standard 16:1 span is predicted to produce an apparent flexural modulus of only 77.6–80.7% of the true longitudinal modulus. The isotropic matrix showed no such loss under identical conditions, establishing that the effect is due to elastic anisotropy and not to the test geometry.
- Extending the span ratio to 32:1 raised the apparent modulus to 93.3–94.3% of the true value.
- The measurement bias is formulation-dependent. Because r-GNFs raise the longitudinal modulus more than the axial shear modulus, the anisotropy ratio increases with filler content, and a 16:1 span is predicted to underreport the true modulus gain at the optimum 0.3 wt% loading by 13.3% of the gain. At 32:1, this falls to 3.9%. Below 0.2 wt%, the bias is negligible, so the distortion is concentrated in the best-performing formulations.
- A rule-of-mixtures decomposition of the source bundle-composite data shows a statistically significant stiffness excess only at 0.3 wt%, confirming the reported optimum while indicating that the apparent trend across the remaining loadings is largely attributable to differences in fiber volume fraction between treatment groups.

Recommendations and future work.

For flexural characterization of the system modelled here, over the anisotropy range examined, a span-to-depth ratio of at least 32:1 is recommended, or alternatively, the reported modulus should be corrected using shear-deformable beam theory. Whether the same threshold holds for other anisotropic polymers is to be determined.

Experimental verification of the predicted bias, through three-point bending of the same formulations at both 16:1 and 32:1, together with direct measurement of *G_xz_* by off axis tension or the Iosipescu method (ASTM D5379), would remove the principal assumption in the present card library. Further gains may also be achievable through surface modification of the UHMWPE fiber itself—plasma, corona or chemical etching treatments that raise interfacial adhesion independently of the matrix—and the same analysis extends to any unidirectional system with a comparable longitudinal-to-shear modulus ratio, such as aramid laminates.

## Figures and Tables

**Table 1:** Tensile verification of the orthotropic card library. Specimen 150 × 25 × 2 mm (*A* = 50 mm^2^), axial displacement 0.75 mm over a 150 mm gauge length, giving ε_x_ = 0.500%.

Card	r-GNF [wt%]	*E*_x_ Input [MPa]	σ_x_ Predicted [MPa]	σ_x_ FEA [MPa]	Error [%]	*F*_x_ [N]	*E*_x_ Backcalc. [MPa]	Error [%]	ν_xy_	ν_xz_
m000	0.00	56051.3	280.257	280.31	0.019	14,016	56,064	0.023	0.29617	0.30351
m010	0.10	56,537.7	282.688	282.75	0.022	14,137	56,548	0.018	0.29617	0.30351
m015	0.15	57,138.6	285.693	285.75	0.020	14,288	57,152	0.023	0.29616	0.30350
m020	0.20	57,990.2	289.951	290.01	0.020	14,500	58,000	0.017	0.29619	0.30351
m030	0.30	67,961.5	339.808	339.87	0.018	16,993	67,972	0.015	0.29617	0.30349
m040	0.40	66,196.7	330.983	331.05	0.020	16,552	66,208	0.017	0.29617	0.30349
m050	0.50	56,283.3	281.417	281.48	0.023	14,074	56,296	0.023	0.29619	0.30351

**Table 2:** Mesh convergence of the tensile model, orthotropic composite card m000. The study was run on an orthotropic card because the tensile configuration is used exclusively to verify the composite library; the matrix cards are exercised only in bending.

Element Size [mm]	Elements	Nodes	DOF	*F*_x_ [N]	σ_x_ Average [MPa]	Modulus Deviation [%]	σ_x_ Peak [MPa]	Change in Peak [%]
1.00	7500	42,655	127,965	14,016	280.32	+0.023	320.92	-
0.75	20,400	104,581	313,743	14,016	280.31	+0.023	331.35	+3.25
0.50	60,000	289,909	869,727	14,016	280.31	+0.023	346.53	+4.58
0.25	480,000	2,118,217	6,354,651	14,016	280.31	+0.023	382.02	+10.24

**Table 3:** Matrix three-point bending at both spans. Specimen 150 × 10 × 4 mm; *D* = 1.000 mm applied to the loading nose. “Within s.d.” tests whether |*E*_app_ − *E*| does not exceed the experimental standard deviation of Ref. [6].

	Card	*E* Measured [MPa]	±s.d.	*P* [N]	σ_max_ [MPa]	E_app_ [MPa]	Error [%]	Within s.d.
L = 64 mm (L/d = 16)	N00	2770	30	27.185	16.377	2783.7	+0.496	yes
	N15	2903	157	28.490	17.163	2917.4	+0.495	yes
	N20	3004	60	29.481	17.760	3018.9	+0.494	yes
	N30	3337	57	32.749	19.729	3353.5	+0.494	yes
	N50	3269	102	32.082	19.327	3285.2	+0.495	yes
L = 128 mm (L/d = 32)	N00	2770	30	3.3807	4.0778	2769.5	−0.019	yes
	N15	2903	157	3.5430	4.2736	2902.4	−0.020	yes
	N20	3004	60	3.6663	4.4222	3003.4	−0.019	yes
	N30	3337	57	4.0727	4.9125	3336.4	−0.019	yes
	N50	3269	102	3.9897	4.8124	3268.4	−0.020	yes

**Table 4:** Mesh convergence of the bending configuration, composite card m030 at *L* = 64 mm. *D*_0_ is the offset in the load-displacement line, defined in Section 5.3. The path runs across the width of the lower face at midspan; its centre and free-edge values are given separately. Changes in E_app_ are relative to the preceding, coarser mesh.

Element Size [mm]	*P* [N]	*E*_app_ [MPa]	Change [%]	*D*_0_ [μm]	Path, Centre [MPa]	Path, Free Edge [MPa]	Whole-Body Max [MPa]	Compressive Extremum [MPa]
2.00	516.24	52,863	-	35.47	307.59	307.85	307.85	−314.25
1.00	515.11	52,747	−0.219	9.37	337.34	337.67	337.67	−376.56
0.75	515.07	52,743	−0.008	3.68	345.66	346.37	346.37	−411.02
0.50	513.40	52,572	−0.324	2.53	350.61	351.74	351.74	−452.14

**Table 5:** Matrix versus composite under identical geometry, contacts, mesh, and loading. Ranges span the five matrix and seven composite cards, respectively.

Quantity	Matrix *L*/*d* = 16	Matrix *L*/*d* = 32	Composite *L*/*d* = 16	Composite *L*/*d* = 32
*E*/*G* ratio	2.70	2.70	50.85–60.81	50.85–60.81
Shear fraction of deflection [%]	1.25	0.32	19.25–22.18	5.62–6.65
*E*_app_/*E* [%]	100.49–100.50	99.98	77.61–80.70	93.26–94.33
Modulus loss [%]	none	none	19.30–22.39	5.67–6.74
Agreement with Timoshenko [%]	+1.77	+0.30	−0.26 to −0.07	−0.16 to −0.05

**Table 6:** True versus reported r-GNF modulus gain, referenced to the 0 wt% card. Because every entry is a ratio of moduli, systematic factors common to all runs cancel exactly.

Card	r-GNF [wt%]	True Gain [%]	Reported at *L*/*d* = 16 [%]	Reported at *L*/*d* = 32 [%]	Loss at 16 [Points]	Loss at 32 [Points]
m000	0.00	0.00	0.00	0.00	-	-
m010	0.10	0.87	0.93	0.89	−0.06	−0.02
m015	0.15	1.94	2.00	1.88	−0.06	+0.06
m020	0.20	3.46	3.55	3.48	−0.09	−0.02
m030	0.30	21.25	18.42	20.41	+2.83	+0.84
m040	0.40	18.10	16.01	17.40	+2.09	+0.70
m050	0.50	0.41	1.97	0.86	−1.55	−0.44

**Table 7:** Sensitivity of the principal results to the assumed fibre axial shear modulus *G*_f_, the one Tier 3 quantity on which the central finding depends. The range is a parametric sweep, not a set of literature values. Each row propagates Gf through the Halpin-Tsai relation to all seven cards and evaluates the Timoshenko solution; ranges span the card library. The true gain, 21.25 points, is set by *E*_x_ alone and is independent of *G*_f_.

*G*_f_ [MPa]	Multiplier	*G*_xz_ Range [MPa]	*E*_x_/*G*_xz_	*E*_app_/*E*_x_ at *L*/*d* = 16 [%]	At *L*/*d* = 32 [%]	Gainunder Reported at 16:1 [Points]	As % of Gain
300	0.29 × baseline	561–628	90.7–108.2	66.4–70.2	88.7–90.4	3.13	14.7
512	-	715–788	72.3–86.3	71.2–74.7	90.8–92.2	3.19	15.0
1024	baseline, after [13]	1025–1118	50.8–60.8	77.8–80.8	93.3–94.4	2.70	12.7
2047	-	1470–1612	35.3–42.1	83.5–85.8	95.3–96.0	1.90	9.0
3280	3.2 × baseline	1824–2026	28.1–33.5	86.4–88.4	96.2–96.8	1.39	6.5

## Data Availability

The original contributions presented in this study are included in the article/Supplementary Material. Further inquiries can be directed to the corresponding author.

## Supplementary Materials

The following supporting information can be downloaded at: https://www.mdpi.com/article/10.3390/polym18212647/s1, Table S1. Isotropic matrix cards. Flexural moduli and their standard deviations are the measured values of Ref. [6] (Tier 1). Poisson’s ratio ν and density ρ are standard literature values for cured DGEBA epoxy (Tier 3); neither is reported in the source. The shear modulus is derived from the isotropic relation G = E/[2(1 + ν)] (Tier 2), giving a constant E/G ratio of 2.700 for every card.; Table S2. Orthotropic composite cards. The longitudinal modulus follows from Equation (5) of the manuscript. The transverse and shear moduli follow from the Halpin-Tsai relations [12] with the fibre properties of Table S4. Cards m010 and m040 use PCHIP-interpolated values of Em(w) and D(w); card m015 uses the measured matrix modulus with an interpolated residual.; Table S3. Provenance and tier of every material constant.; Table S4. Inputs used in the construction of the composite cards.

## Abbreviations

The following abbreviations are used in this manuscript:

ANSYS	Analysis System (commercial finite element software)
ASTM	American Society for Testing and Materials
CI	Confidence interval
DGEBA	Diglycidyl ether of bisphenol A
DOF	Degrees of freedom
FEA	Finite element analysis
GCNF	Graphitic carbon nanofiber
GNF	Graphitic nanofiber
ISO	International Organization for Standardization
PCHIP	Piecewise cubic Hermite interpolating polynomial
r-GNF	Reactive graphitic nanofiber
STEP	Standard for the Exchange of Product Data
UHMWPE	Ultra-high-molecular-weight polyethylene
