# Supplementary material for: Evaluating Measurement Bias in Flexural Modulus of Orthotropic UHMWPE Fiber/Nano-Epoxy Composites: A Finite Element Study
Source: Polymers (Basel). 2026 Oct 26;18(21):2647. doi: 10.3390/polym18212647 (PMC13766172; doi:10.3390/polym18212647)
Supplement: Supplementary file 1 [file polymers-18-02647-s001.zip › polymers-4592525-Supplementary.pdf]

Supplementary Material S1

Material card library and data provenance

Evaluating Measurement Bias in Flexural Modulus of Orthotropic UHMWPE Fiber/Nano-Epoxy Composites: A Finite Element Study

1. Tier classification

All material constants are classified into three tiers according to provenance. Tier 1 values are taken directly from measurement; Tier 2 values are derived from Tier 1 data using published relations; Tier 3 values are literature assumptions. The classification of every constant is given in Table S3.

2. Isotropic matrix cards

Table S1. Isotropic matrix cards. Flexural moduli and their standard deviations are the measured values of Ref. [6] (Tier 1). Poisson's ratio ν and density ρ are standard literature values for cured DGEBA epoxy (Tier 3); neither is reported in the source. The shear modulus is derived from the isotropic relation G = E / [2(1 + ν)] (Tier 2), giving a constant E/G ratio of 2.700 for every card.

Card	r-GNF [wt%]	E [MPa]	s.d. [MPa]	Poisson's ratio ν	Density ρ [kg m⁻³]	G [MPa]
N00	0.00	2770	30	0.35	1150	1025.9
N15	0.15	2903	157	0.35	1150	1075.2
N20	0.20	3004	60	0.35	1150	1112.6
N30	0.30	3337	57	0.35	1150	1235.9
N50	0.50	3269	102	0.35	1150	1210.7

3. Orthotropic composite cards

Table S2. Orthotropic composite cards. The longitudinal modulus follows from Equation (5) of the manuscript. The transverse and shear moduli follow from the Halpin-Tsai relations [12] with the fibre properties of Table S4. Cards m010 and m040 use PCHIP-interpolated values of Em(w) and D(w); card m015 uses the measured matrix modulus with an interpolated residual.

Card	r-GNF w [wt%]	Em [MPa]	D(w) [MPa]	Ex [MPa]	Ey = Ez	Gxy = Gxz	Gyz	νxy = νxz	νyz	Ex/Gxz
m000	0.00	2770.00	0.00	56051.2	5587.3	1024.9	1991.2	0.302	0.403	54.69
m010	0.10	2839.64	454.13	56537.7	5658.6	1036.8	2016.6	0.302	0.403	54.53
m015	0.15	2903.00	1025.57	57138.6	5722.3	1047.5	2039.3	0.302	0.403	54.55
m020	0.20	3004.00	1830.20	57990.2	5821.7	1064.3	2074.7	0.302	0.403	54.49
m030	0.30	3337.00	11646.66	67961.5	6132.3	1117.8	2185.4	0.302	0.403	60.80
m040	0.40	3328.50	9885.81	66196.7	6124.6	1116.5	2182.7	0.302	0.403	59.29
m050	0.50	3269.00	0.00	56283.2	6070.8	1107.1	2163.5	0.302	0.403	50.84

All moduli in MPa. Computed values agree with Table 1 of the manuscript to within 0.1 MPa on Ex and 0.01 on Ex/Gxz; the residual reflects rounding of the inputs quoted in the text and has no effect on any reported result.

4. Provenance and tier classification

Table S3. Provenance and tier of every material constant.

Constant	Applies to	Source / relation	Tier	Note
E(w)	Matrix cards	Measured flexural modulus, Figure 2 of [6]	1	Five values; the entire matrix library.
ν	Matrix cards	Literature value for cured DGEBA epoxy	3	Not reported in the source.
ρ	Matrix cards	Literature value for cured DGEBA epoxy	3	Not reported in the source.

Constant	Applies to	Source / relation	Tier	Note
G	Matrix cards	G = E / [2(1 + ν)]	2	Isotropic relation; E/G = 2.700 throughout.
Ef	Composite cards	Least-squares fit to the ten pure-epoxy bundle specimens of [7]	2	102 361 MPa, 95% CI [98.0, 106.7] GPa.
Vf	Composite cards	Chosen within the envelope of three of the four groups of [7]	3	Fixed at 0.535; justified in Section 2.2.2.
Em(w)	Composite cards	Measured flexural modulus from [6]	1	Cards m010 and m040 interpolated by PCHIP.
D(w)	Composite cards	Residual of the rule of mixtures against the bundle data of [7]	2	Significant only at 0.30 wt% after Holm-Bonferroni correction.
Ex	Composite cards	Equation (5)	2	Combines Tier 1 and Tier 2 inputs.
Ey, Ez	Composite cards	Halpin-Tsai [12], xi = 2, fibre transverse modulus after [13]	3	Fibre value assumed, not measured.
Gxy, Gxz	Composite cards	Halpin-Tsai [12], xi = 1, Gf = 1024 MPa after [13]	3	Principal assumption; sensitivity in Section 5.2.
Gyz	Composite cards	Gyz = Ey / [2(1 + νyz)]	3	Follows from Ey, itself Tier 3.
νxy, νxz, νyz	Composite cards	Rule of mixtures	2	Constant across formulations.
ρ	Composite cards	Volumetric rule of mixtures	2	Constant at 1054 kg m^{-3}.

5. Calibration inputs

Table S4. Inputs used in the construction of the composite cards.

Quantity	Value	Unit	Source
Fibre volume fraction Vf	0.535	-	Chosen; Section 2.2.2
Fibre longitudinal modulus Ef	102 361	MPa	Least-squares fit to [7]
Matrix volume fraction Vm	0.465	-	Equals 1 minus Vf
Fibre transverse modulus Eft	10 236	MPa	Chocron convention [13]: one order below Ef
Fibre axial shear modulus Gf	1024	MPa	Chocron convention [13]: two orders below Ef
Halpin-Tsai xi, axial shear	1	-	Circular fibres, square packing [10,12]
Halpin-Tsai xi, transverse	2	-	Circular fibres, square packing [10,12]

Ef and Gf are Tier 3 assumptions transferred from Dyneema HB80; their influence on the results is examined in Section 5.2 of the manuscript.

6. Source references

[6] Jana, S.; Zhong, W.-H.; Gan, Y.X. Mater. Sci. Eng. A 2007, 445-446, 106-112.

[7] Jana, S.; Hinderliter, B.R.; Zhong, W.H. J. Mater. Sci. 2008, 43, 4236-4246.

[10] Jones, R.M. Mechanics of Composite Materials, 2nd ed.; Taylor & Francis: Philadelphia, PA, USA, 1999.

[12] Halpin, J.C.; Kardos, J.L. Polym. Eng. Sci. 1976, 16, 344-352.

[13] Chocron, S.; King, N.; Bigger, R.; Walker, J.D.; Heisserer, U.; van der Werff, H. J. Appl. Mech. 2013, 80, 031806.
